# Goitres and Interstiteal Medication with Iodine

**Published:** 1887-04

**Authors:** 


Goitres and Interstiteal Medication with Iodine.—Dr. Dugent, in a paper in the *Proces Agrege á la faculte Med. de l'Hop. Lariboisière*, Paris, Dec., 1886, concludes that extirpation of the thyroid being followed with such great sequelæ (myxœdema), the treatment should be limited to injections with tincture of iodine, especially as partial extirpation does not forbid a recurrence. The author cites the cure of twenty-one cases out of thirty four treated by him with iodine injections; of the other thirteen, seven cases were ameliorated, the remainder were incomplete. It is not the character of the goitre, cystic, parenchymatous, etc., but the age of the tumors, which will influence the result of the treatment. Old goitres will remain refractory. Tincture of iodine acts, first, by the irritant action of the alcohol, and, second, by the specific action of the iodine. Dr. Dugent has made 266 injections without accident.—*Journal of Laryngology and Rhinology.*

